# Conectando Chamados Globais à Ação Local: Avançando a Equidade Cardiovascular para Mulheres no Brasil

**DOI:** 10.36660/abc.20260110

**Published:** 2026-04-14

**Authors:** Ariane Vieira Scarlatelli Macedo, Fernanda Souza de Carvalho, Teresa Glynn, Neil Johnson, Karen Padilla Cabrera, Marlene Oliveira

**Affiliations:** 1 Faculdade de Ciências Médicas de Santa Casa de São Paulo São Paulo SP Brasil Faculdade de Ciências Médicas de Santa Casa de São Paulo, São Paulo, SP – Brasil; 2 Instituto Lado a Lado pela Vida São Paulo SP Brasil Instituto Lado a Lado pela Vida, São Paulo, SP – Brasil; 3 Global Heart Hub Galway Irlanda Global Heart Hub, Galway – Irlanda

**Keywords:** Doenças Cardiovasculares, Mulheres, Equidade em Saúde, Disparidades em Saúde, Prevenção Primária

O *Global Burden of Disease* 2023 destaca o impacto catastrófico das doenças cardiovasculares, responsáveis por 19,2 milhões de mortes em todo o mundo em 2023. As mulheres apresentam 20% mais mortalidade após infarto agudo do miocárdio, maior prevalência de insuficiência cardíaca com fração de ejeção preservada e riscos metabólicos crescentes (como índice de massa corporal) em comparação aos homens. De forma crítica, 79,6% da carga global de doenças cardiovasculares é atribuível a fatores de risco modificáveis — incluindo hipertensão arterial, dislipidemia, dieta e poluição do ar —, mas as taxas padronizadas por idade permanecem mais altas em contextos de baixa renda, onde as mulheres são desproporcionalmente afetadas.^1,2^

As evidências mostram de forma consistente que as mulheres enfrentam desvantagens específicas no cuidado cardiovascular.^1–3^ Elas têm maior probabilidade de vivenciar doença cardíaca tardia, não diagnosticada ou diagnosticada incorretamente, impulsionada por lacunas no reconhecimento dos sintomas, caminhos diagnósticos centrados nos homens, sub-representação em pesquisas e cuidados fragmentados.^4,5^ Essas desigualdades sistêmicas contribuem para atrasos no diagnóstico e piores resultados de tratamento para as mulheres.

Essas disparidades de sexo no cuidado cardiovascular refletem falhas estruturais e sistêmicas de longa data que exigem ação urgente e coordenada. Dados de coortes de longo prazo mostram que uma posição socioeconômica mais baixa está fortemente associada a transições mais precoces da boa saúde para multimorbidade, fragilidade e morte, ressaltando que os ganhos em equidade dependem da prevenção primária e da detecção precoce, e não apenas de intervenções em estágios avançados.^6^ Pacientes e cuidadores são parceiros essenciais na identificação dessas lacunas.

Na seção "*Comment*" na revista *The Lancet Regional Health* – *Europe, Padilla Cabrera e Johnson, da Global Heart Hub (GHH)*, uma das alianças internacionais de organizações de pacientes cardíacos mais ativas e amplamente reconhecidas, descrevem como a distância geográfica, as dificuldades financeiras, o viés de gênero e as normas culturais restringem o acesso à prevenção, à detecção precoce e ao atendimento especializado.^6^ As mulheres relatam que os caminhos de cuidado raramente acomodam suas responsabilidades de cuidado, limitações econômicas e necessidades de saúde mental.^7^

Esses relatórios validam os achados epidemiológicos e reforçam uma mensagem-chave: enfrentar as desigualdades cardiovasculares exige não apenas soluções técnicas, mas também um engajamento significativo com os pacientes para delinear cuidados culturalmente apropriados e centrados no indivíduo. As alianças globais de pacientes são atores críticos nesse esforço. Por meio de cúpulas internacionais, pesquisas lideradas por pacientes e mesas-redondas nacionais, essas alianças ajudaram a redefinir a doença cardiovascular (DCV), deixando de ser vista apenas como um desafio clínico para ser reconhecida como uma questão de equidade — na qual as experiências vividas por mulheres com condições cardiovasculares orientam as prioridades de ação.^4,7,8^

O Brasil alcançou reduções substanciais na mortalidade por DCV nas últimas décadas, mas esses avanços foram profundamente desiguais entre regiões e grupos sociais.^9,10^ Estados com indicadores sociais mais precários – particularmente no Norte e Nordeste – apresentaram quedas mais lentas na mortalidade por doenças circulatórias, refletindo disparidades persistentes nos determinantes sociais, na infraestrutura de saúde e no acesso ao cuidado.^9,10^

Para as mulheres brasileiras, a carga dos principais fatores de risco cardiovascular permanece elevada e fortemente estratificada por educação, renda e raça/cor da pele.^11,12^ Experiências de discriminação racial têm sido associadas a marcadores adversos de risco cardiovascular, sugerindo que o racismo atua como um determinante fundamental da inequidade em saúde cardiovascular.^11^ Fatores de risco específicos e relacionados ao sexo acrescentam maior complexidade: distúrbios hipertensivos da gestação, diabetes gestacional e outros desfechos adversos da gravidez são importantes marcadores de risco cardiovascular futuro, embora continuem sub-reconhecidos e subdocumentados na prática clínica.^11^ A mortalidade materna permanece inaceitavelmente alta, especialmente no Norte e Nordeste, afetando de forma desproporcional mulheres negras e indígenas – evidenciando como riscos reprodutivos e cardiovasculares se entrelaçam em contextos de vulnerabilidade social.^11^

A reabilitação cardíaca e a prevenção secundária ilustram como as desigualdades se acumulam ao longo dos caminhos de cuidado. Evidências internacionais mostram que mulheres e grupos socioeconomicamente desfavorecidos estão consistentemente sub-representados em programas de reabilitação, apesar dos claros benefícios clínicos e psicológicos.^7^ Os dados brasileiros apontam para desafios semelhantes, com disponibilidade limitada de programas fora dos grandes centros urbanos e barreiras relacionadas ao transporte, tempo, responsabilidades de cuidado e custos diretos.^11,13^ Os marcos globais sobre prevenção de DCV e equidade fornecem orientações essenciais, mas não podem ser aplicados ao Brasil de forma padronizada ("*one-size-fits-all*").^1,3^

A organização do sistema de saúde é um determinante crucial: o amplo sistema público universal do Brasil (Sistema Único de Saúde, SUS) coexiste com um setor privado segmentado e marcada heterogeneidade regional em infraestrutura, força de trabalho e capacidade de implementar intervenções complexas.^9,10^ Recomendações que pressupõem acesso universal a diagnósticos avançados, acompanhamento contínuo com especialistas ou programas intensivos de reabilitação podem, inadvertidamente, ampliar desigualdades se sua implementação permanecer concentrada em centros urbanos mais ricos e entre populações com seguro de saúde.^6,10^

Uma segunda limitação é a disponibilidade de dados para monitorar desigualdades sob uma lente interseccional. Embora as estatísticas nacionais relatem cada vez mais indicadores cardiovasculares desagregados por sexo e região, a desagregação sistemática por raça/etnia, renda e outras dimensões relevantes permanece incompleta.^11–13^ Muitos aspectos do cuidado cardiovascular das mulheres — como acesso à reabilitação, qualidade da prevenção secundária, integração entre saúde reprodutiva e cardiovascular, e experiências de discriminação nos serviços de saúde — não são rotineiramente capturados nos sistemas de informação.^11–13^

O Brasil oferece um exemplo convincente de como organizações de pacientes e a sociedade civil podem impulsionar mudanças estruturais na política de saúde. O instituto Lado a Lado pela Vida (LAL), afiliado à GHH, tem estado na linha de frente da defesa tanto da saúde cardiovascular quanto da saúde oncológica desde 2008.^14^ O LAL participou de todas as etapas que levaram à aprovação da Lei 14.758/2023, que instituiu a Política Nacional de Prevenção e Controle do Câncer (PNPCC).^14^ Essa legislação histórica, a primeira política abrangente de câncer no país, tem como objetivo reduzir a incidência da doença, garantir acesso ao cuidado integrado, melhorar a qualidade de vida e reduzir a mortalidade e a incapacidade causadas pelo câncer.^14^

A PNPCC foi resultado de uma colaboração contínua entre a Comissão Especial de Controle do Câncer da Câmara dos Deputados, organizações de pacientes, sociedades médicas e a sociedade civil. O papel do LAL exemplifica como organizações do terceiro setor podem avançar da defesa de interesses para a co-criação de políticas públicas: mobilizando pacientes, engajando legisladores, contribuindo para a redação de textos legais e mantendo pressão pela implementação efetiva.^14^

O Brasil está bem posicionado para servir como modelo no avanço da equidade em saúde cardiovascular para mulheres, exemplificando como recomendações globais podem ser concretizadas por meio de parcerias locais. A combinação de um sistema de saúde universal, sociedades de cardiologia fortes e uma defesa organizada de pacientes oferece um contexto favorável para ações coordenadas.^11,15^ A recente colaboração entre a GHH e o LAL no Brasil — incluindo uma mesa-redonda multissetorial sobre a integração das perspectivas dos pacientes na política cardiovascular — ilustra como iniciativas globais podem enriquecer o diálogo local e apoiar o desenvolvimento de uma estratégia nacional de DCV que incorpore explicitamente equidade e experiência do paciente.^14^ Um roteiro prático para o Brasil poderia incluir três linhas de ação complementares.

Primeiro, a pesquisa e a vigilância devem incorporar sistematicamente uma lente de equidade. Pesquisas nacionais, bases de dados administrativas e registros clínicos precisam priorizar análises desagregadas por sexo, raça/cor da pele, renda e região, além de incluir indicadores relevantes para a saúde cardiovascular das mulheres — como complicações relacionadas à gravidez e acesso à reabilitação —, incentivando a colaboração com defensores de pacientes e a sociedade civil na definição das perguntas de pesquisa e na interpretação dos resultados.^11,13^

Segundo, a detecção precoce e a atenção primária devem ser fortalecidas de maneiras social e culturalmente sensíveis. O manifesto da GHH para detecção e diagnóstico precoce de DCV enfatiza a necessidade de redesenhar os caminhos de cuidado, aproveitar as tecnologias digitais e expandir a capacitação da força de trabalho para garantir o diagnóstico oportuno das condições cardiovasculares mais comuns.^4,7^ No Brasil, traduzir esses princípios requer reforçar a capacidade das equipes de atenção primária do SUS (Estratégia Saúde da Família) para identificar e manejar mulheres em maior risco cardiovascular ao longo da vida, integrar saúde reprodutiva e cardiovascular, e utilizar agentes comunitários de saúde e telessaúde para alcançar mulheres em áreas remotas ou desassistidas.^11,13^

Terceiro, a prevenção secundária, a reabilitação cardíaca e o cuidado integrado em saúde mental devem ser redesenhados para responder às necessidades e restrições específicas das mulheres. Isso inclui enfrentar barreiras logísticas como agendamento, transporte e responsabilidades de cuidado; explorar modelos comunitários e domiciliares; e incorporar avaliação psicológica e apoio no cuidado cardiovascular de rotina.^4,7^

Em todos esses domínios, as estruturas de governança devem avançar da consulta para a partilha significativa de poder com os pacientes. A Sociedade Brasileira de Cardiologia (SBC) e seu Departamento de Cardiologia da Mulher, juntamente com outros atores nacionais, podem estabelecer painéis permanentes de aconselhamento de pacientes, incluir representantes de pacientes em comitês de diretrizes e posicionamentos, e incorporar medidas de desfecho e experiência relatadas pelos pacientes nos indicadores de qualidade do cuidado.^4,14,15^

Fechar as lacunas de inequidade na saúde cardiovascular das mulheres brasileiras não será alcançado apenas com a adesão a declarações globais ou pela adaptação de diretrizes internacionais. Isso requer a tradução deliberada de visões globais em políticas nacionais, prioridades de pesquisa e modelos de cuidado que enfrentem as interseções específicas de sexo, raça/cor da pele, classe e território que moldam a vida das mulheres no Brasil.^6,11^ O sucesso da defesa do LAL pela Política Nacional do Câncer demonstra que parcerias sustentadas e estruturadas entre organizações de pacientes, sociedades médicas e formuladores de políticas podem produzir legislação transformadora e, em última instância, melhores resultados para os pacientes.^14^

Organizações de pacientes, como GHH e LAL, juntamente com a SBC, profissionais de saúde, gestores e formuladores de políticas públicas, compartilham a responsabilidade de garantir que a equidade cardiovascular se torne um resultado mensurável, e não apenas um princípio declarado.^4,7^ A comunidade global de pacientes com doenças cardiovasculares tem transmitido uma mensagem clara: o valor da experiência vivida e dos dados relatados pelos pacientes é indiscutível, e os pacientes estão prontos para participar ativamente de ações voltadas para a equidade.^7^

Para a cardiologia brasileira, o próximo passo é incorporar essa mensagem de forma estrutural, integrando as vozes dos pacientes ao centro da tomada de decisões. Somente ao combinar perspectivas globais com estratégias localmente fundamentadas e centradas no paciente — e ao reconhecer as mulheres como coautoras da mudança — será possível garantir que nenhum coração, e nenhum coração de mulher, seja deixado para trás na atenção cardiovascular brasileira.
