# Supplementary material for: Conectando Chamados Globais à Ação Local: Avançando a Equidade Cardiovascular para Mulheres no Brasil
Source: Arq Bras Cardiol. 2026 Apr 14;123(3):e20260110. [Article in Portuguese] doi: 10.36660/abc.20260110 (PMC13128210; doi:10.36660/abc.20260110)
Supplement: Translation

# Translation: Bridging Global Calls to Local Action: Advancing Cardiovascular Equity for Women in Brazil

**DOI:** 10.36660/abc.20260110i

**Authors:** Ariane Vieira Scarlatelli Macedo, Fernanda Souza de Carvalho, Teresa Glynn, Neil Johnson, Karen Padilla Cabrera, Marlene Oliveira

**Affiliations:** 1 São Paulo SP Brazil Faculdade de Ciências Médicas de Santa Casa de São Paulo, São Paulo, SP – Brazil; 2 São Paulo SP Brazil Instituto Lado a Lado pela Vida, São Paulo, SP – Brazil; 3 Galway Ireland Global Heart Hub, Galway – Ireland

**Keywords:** Cardiovascular Diseases, Women, Health Equity, Health Status Disparities, Primary Prevention

The Global Burden of Disease 2023 highlights the catastrophic impact of cardiovascular disease, accounting for 19.2 million deaths worldwide in 2023.^1^ Women experience 20% higher post-myocardial infarction mortality, a greater prevalence of heart failure with preserved ejection fraction, and faster-rising metabolic risks (such as body mass index) compared with men. Critically, 79.6% of the global cardiovascular burden is attributable to modifiable risk factors – including high blood pressure, dyslipidemia, diet, and air pollution – yet age-standardized rates remain highest in low-income settings, where women are disproportionately affected.^1,2^

Evidence consistently shows that women face specific disadvantages across the cardiovascular care continuum.^1–3^ They are more likely to experience late, missed, or misdiagnosed heart disease, driven by gaps in symptom recognition, male-centered diagnostic pathways, underrepresentation in research, and fragmented care.^4,5^ These systemic inequities contribute to delayed diagnosis and poorer treatment outcomes for women.

These sex disparities in cardiovascular care reflect long-standing structural and systemic failures that demand urgent, coordinated action. Long-term cohort data show that lower socioeconomic position is strongly associated with earlier transitions from good health to multimorbidity, frailty, and death, underscoring that equity gains depend on primary prevention and early detection rather than late-stage interventions alone.^6^ Patients and caregivers are essential partners in identifying these gaps.

In their Comment in The Lancet Regional Health – Europe, Padilla Cabrera and Johnson, from Global Heart Hub (GHH)—one of the most active and widely recognized international alliances of heart patient organizations—describe how geographic distance, financial hardship, gender bias, and cultural norms restrict access to prevention, early detection, and specialist care.^6^ Women report that care pathways rarely accommodate their caregiving responsibilities, economic constraints, and mental health needs.^7^

These reports validate epidemiological findings and reinforce a key message: tackling cardiovascular inequities requires not only technical solutions but also meaningful engagement with patients to co-design culturally appropriate, person-centered care. Global patient alliances are critical actors in this effort. Through international summits, patient-led research, and national roundtables, these alliances have helped reframe Cardiovascular Disease (CVD) from being viewed solely as a clinical challenge to a broader equity issue – where the lived experiences of women with cardiovascular conditions guide priorities for action.^4,7,8^

Brazil has achieved substantial reductions in CVD mortality over recent decades, but these gains have been profoundly unequal across regions and social groups.^9,10^ States with poorer social indicators – particularly in the North and Northeast – have experienced slower declines in circulatory disease mortality, reflecting persistent disparities in social determinants, health infrastructure, and access to care.^9,10^

For Brazilian women, the burden of major cardiovascular risk factors remains high and is strongly stratified by education, income, and race/skin color.^11,12^ Experiences of racial discrimination have been linked to adverse cardiovascular risk markers, suggesting that racism functions as a fundamental determinant of cardiovascular health inequity.^11^ Sex-specific and sex-related risk factors add further complexity: hypertensive disorders of pregnancy, gestational diabetes, and other adverse pregnancy outcomes are important markers of future cardiovascular risk, yet they remain under-recognized and under-documented in routine care.^11^ Maternal mortality remains unacceptably high, particularly in the North and Northeast, and disproportionately affects Black and Indigenous women, highlighting how reproductive and cardiovascular risks intersect in contexts of social vulnerability.^11^

Cardiac rehabilitation and secondary prevention illustrate how inequities accumulate along care pathways. International evidence shows that women and socioeconomically disadvantaged groups are consistently underrepresented in rehabilitation programs, despite clear clinical and psychological benefits.^7^ Brazilian data point to similar challenges, with limited availability of programs outside major urban centers and barriers related to transport, time, caregiving responsibilities, and out-of-pocket costs.^11,13^ Global frameworks on CVD prevention and equity provide essential guidance, but they cannot be applied to Brazil in a "one-size-fits-all" manner.^1,3^

Health-system organization is a crucial determinant: Brazil's large universal public system (Sistema Único de Saúde, SUS) coexists with a segmented private sector and marked regional heterogeneity in infrastructure, workforce, and capacity to implement complex interventions.^9,10^ Recommendations that presuppose universal access to advanced diagnostics, continuous specialist follow-up, or intensive rehabilitation programs may inadvertently widen disparities if their implementation remains concentrated in wealthier urban centers and among insured populations.^6,10^

A second limitation is the availability of data to monitor inequities through an intersectional lens. Although national statistics increasingly report cardiovascular indicators disaggregated by sex and region, systematic disaggregation by race/ethnicity, income, and other relevant dimensions remains incomplete.^11–13^ Many aspects of women's cardiovascular care – such as access to rehabilitation, quality of secondary prevention, integration of reproductive and cardiovascular health, and experiences of discrimination in health services – are not routinely captured in information systems.^11–13^

Brazil offers a compelling example of how patient organizations and civil society can drive structural change in health policy. The *Lado a Lado pela Vida* (LAL) institute, a Global Heart Hub (GHH) affiliate, has been at the forefront of advocacy for both cancer and cardiovascular health since 2008.^14^ LAL participated in every stage leading to the approval of Law 14.758/2023, which established Brazil's National Policy for Cancer Prevention and Control (PNPCC).^14^ This landmark legislation, the country's first comprehensive cancer policy, aims to reduce cancer incidence, guarantee access to integrated care, improve quality of life, and lower mortality and disability caused by cancer.^14^

The PNPCC was the result of sustained collaboration between the Special Commission for Cancer Control in the Chamber of Deputies, patient organizations, medical societies, and civil society. LAL's role exemplifies how third-sector organizations can move from advocacy to co-creation of public policy: mobilizing patients, engaging legislators, contributing to the drafting of legal texts, and maintaining pressure for effective implementation.^14^

Brazil is well positioned to serve as a model for advancing equitable cardiovascular health for women, exemplifying how global recommendations can be realized through local partnerships. The combination of a universal health system, strong cardiology societies, and organized patient advocacy provides a favorable context for coordinated action.^11,15^ The recent collaboration between GHH and LAL in Brazil – including a multistakeholder roundtable on integrating patient perspectives into cardiovascular policy – illustrates how global initiatives can enrich local dialogue and support the development of a national CVD strategy that explicitly incorporates equity and patient experience.^14^ A practical roadmap for Brazil could include three complementary lines of action.

First, research and surveillance should systematically incorporate an equity lens. National surveys, administrative databases, and clinical registries must prioritize analyses disaggregated by sex, race/skin color, income, and region, and should include indicators relevant to women's cardiovascular health, such as pregnancy-related complications and access to rehabilitation, while encouraging collaboration with patient advocates and civil society in defining research questions and interpreting results.^11,13^

Second, early detection and primary care must be strengthened in ways that are socially and culturally attuned. The GHH manifesto for early detection and diagnosis of CVD emphasizes redesigning care pathways, leveraging digital technologies, and expanding workforce training to ensure timely diagnosis of common cardiovascular conditions.^4,7^ In Brazil, translating these principles requires reinforcing the capacity of SUS primary care teams (*Saúde da Família* strategy) to identify and manage women at increased cardiovascular risk across the life course, to integrate reproductive and cardiovascular health, and to use community health workers and telehealth to reach women in remote or underserved areas.^11,13^

Third, secondary prevention, cardiac rehabilitation, and integrated mental health care should be redesigned to respond to women's specific needs and constraints. This includes addressing logistical barriers such as scheduling, transportation, and caregiving responsibilities; exploring community-based and home-based models; and embedding psychological assessment and support in routine cardiovascular care.^4,7^

Across all these domains, governance structures must move from consultation to meaningful power-sharing with patients. The Brazilian Society of Cardiology (SBC) and its Department of Women's Cardiology, together with other national stakeholders, can establish permanent patient advisory panels, include patient representatives in guideline and position-statement committees, and incorporate patient-reported outcome and experience measures into quality-of-care indicators.^4,14,15^

Closing inequity gaps in cardiovascular health for Brazilian women will not be achieved solely by endorsing global declarations or adapting international guidelines. It requires deliberate translation of global visions into national policies, research priorities, and care models that confront the specific intersections of sex, race/skin color, class, and territory that shape women's lives in Brazil.^6,11^ The success of LAL's advocacy for the National Cancer Policy demonstrates that sustained, structured partnerships between patient organizations, medical societies, and policymakers can produce transformative legislation and, ultimately, better outcomes for patients.^14^

Patient organizations such as GHH and LAL, together with the Brazilian Society of Cardiology, health professionals, managers, and policymakers, share responsibility for ensuring that cardiovascular equity becomes a measurable outcome rather than merely a stated principle.^4,7^ The global CVD patient community has articulated a clear message: the value of lived experience and patient-reported data is indisputable, and patients stand ready to co-design equity-driven action.^7^

For Brazilian cardiology, the next step is to embed this message structurally, integrating patient voices into the heart of decision-making. Only by combining global perspectives with locally grounded, patient-centered strategies – and by recognizing women as co-authors of change – will it be possible to ensure that no heart, and no woman's heart, is left behind in Brazilian cardiovascular care.
